# Neural Underpinnings of Decision Strategy Selection: A Review and a Theoretical Model

**DOI:** 10.3389/fnins.2016.00500

**Published:** 2016-11-08

**Authors:** Szymon Wichary, Tomasz Smolen

**Affiliations:** ^1^Unit for Psychophysiology of Cognitive Processes, Department of Psychology, SWPS University of Social Sciences and HumanitiesWarsaw, Poland; ^2^Department of Psychology, Pedagogical University of KrakowKrakow, Poland

**Keywords:** strategy selection, multi-attribute choice, decision-making, neurocognitive model, arousal, gain modulation

## Abstract

In multi-attribute choice, decision makers use decision strategies to arrive at the final choice. What are the neural mechanisms underlying decision strategy selection? The first goal of this paper is to provide a literature review on the neural underpinnings and cognitive models of decision strategy selection and thus set the stage for a neurocognitive model of this process. The second goal is to outline such a unifying, mechanistic model that can explain the impact of noncognitive factors (e.g., affect, stress) on strategy selection. To this end, we review the evidence for the factors influencing strategy selection, the neural basis of strategy use and the cognitive models of this process. We also present the Bottom-Up Model of Strategy Selection (BUMSS). The model assumes that the use of the rational Weighted Additive strategy and the boundedly rational heuristic Take The Best can be explained by one unifying, neurophysiologically plausible mechanism, based on the interaction of the frontoparietal network, orbitofrontal cortex, anterior cingulate cortex and the brainstem nucleus locus coeruleus. According to BUMSS, there are three processes that form the bottom-up mechanism of decision strategy selection and lead to the final choice: (1) cue weight computation, (2) gain modulation, and (3) weighted additive evaluation of alternatives. We discuss how these processes might be implemented in the brain, and how this knowledge allows us to formulate novel predictions linking strategy use and neural signals.

Adaptive decision making requires processing of information from various sources. Do we integrate multiple decision cues or process them selectively? The use of various strategies of cue processing helps us make decisions flexibly, sometimes extensively analyzing information, and simplifying the decision process at other times. What are the neural mechanisms of decision strategy selection? Can we explain the impact of emotional factors on strategy use by understanding its neural underpinnings? The first goal of this paper is to provide a literature review on the models and neural correlates of decision strategy selection and thus set the stage for a unifying neurocognitive model of this process. The second goal is to outline such a model, that is able to explain affective influences on strategy selection.

In multi-attribute choice, people decide which alternative they choose out of several available choice alternatives, after they judge the alternatives on one or more attributes (or cues). This kind of choice tasks may be further divided into preferential choices and probabilistic inferences (Payne et al., 1993; Gigerenzer et al., 1999). In preferential choices, the criterion that the individuals maximize is subjective—for example, a person might decide between two dishes in a restaurant, imagining how tasty they are. In contrast, choices based on probabilistic inference deal with objective criterion values. For example, paramedics may use several decision cues to infer and decide which patient must be treated first in an accident, or stock brokers might decide in which stocks to invest after analyzing companies' performance over the previous year. The cues are probabilistically related to the criterion, so a positive cue value makes a positive criterion more likely. Multi-attribute choices require a relatively long sequence of cognitive operations. Classical theories of choice postulated that when making such choices, humans should process all available information, carefully weighing the attributes, multiplying the weights by the attribute values and adding the products of this multiplication in order to arrive at alternatives' overall judgment which then can be compared (Keeney and Raiffa, 1993). This has been known as the weighted additive (WADD) model (Payne et al., 1993). As alternatives to this normative view, descriptive approaches stress that people frequently use heuristics to simplify decision problems (Payne et al., 1993; Gigerenzer et al., 1999; Table 1). A lexicographic strategy Take The Best (TTB) is a flagship example of simple heuristics for multi-attribute choice. It infers that when comparing two alternatives, the one with the highest value on the most valid attribute is selected as the one with the highest criterion value. If the cue with the highest validity does not discriminate, then Take The Best considers the cue with the second highest validity, and so on (Gigerenzer and Goldstein, 1996).

**Table 1:** **Decision strategies for multi-attribute choice with references to empirical studies**.

**Strategy**	**Studies**	**Description**
Weighted additive WADD	Payne et al., 1993	Choice alternatives (e.g., cars, houses) can be described by sets of attributes or cues (e.g., color, price) which have values (e.g., red, blue or 10,000, 20,000 $) and weights (subjectively or objectively predetermined attribute importance). Weighted additive calculates for each alternative the sum of the attribute values multiplied by the corresponding cue weights and selects the alternative with the highest score.
Equal weight; additive; unit weight ADD	Dawes, 1979	Calculates for each alternative the sum of the cue values (multiplied by a weight of 1) and selects the alternative with the highest score.
Elimination by aspects EBA	Tversky, 1972	Eliminates all alternatives that do not exceed a specified value on the first cue examined. If more than one alternative remains, another cue is considered. This procedure is repeated until only one alternative is left. Each cue is selected with a probability proportional to its weight.
Lexicographic model LEX	Fishburn, 1974	Selects the alternative with the highest cue value on the cue with the highest validity. If more than one alternative has the same highest cue value, then for these alternatives the cue with the second highest validity is considered, and so on.
Lexicographic semiorder LEX-SEMI	Luce, 1956	Works like Lexicographic, with the additional assumption of a negligible difference. Pairs of alternatives with a negligible difference between the cue values are not discriminated.
Take The Best TTB	Gigerenzer and Goldstein, 1996	A special case of Lexicographic for two alternatives with binary cue values (e.g., “rains today,” “does not rain today”). Cue validity can be used as cue weight in this case—it is a conditional probability of the cue's success in predicting the criterion value (e.g., predicting if it will rain tomorrow based on today's weather) given that the cue discriminates among the alternatives (alterantives do not have the same cue values).

Choice strategies differ in the effort to execute them. For example, WADD always processes all available decision cues and multiplies their values by their weights. Strategies like WADD integrate information into alternatives' overall judgments. These strategies are called compensatory, because low values on one attribute can be compensated by high values on other attributes (Dawes and Corrigan, 1974). In turn, simple strategies like TTB do not integrate information—their decisions are based on a single cue, the rest of the information is ignored. Such strategies are thus called noncompensatory, because values of the less important cues cannot compensate for the cue value of the most important cue (Einhorn, 1970). Given their simplicity, simple heuristics have been proposed as plausible models of predecisional information processing and choice (Payne et al., 1993; Gigerenzer et al., 1999).

## Factors influencing strategy use

Strategy use is influenced by various dispositional and situational factors. Early studies identified that learning related factors—expertise, training, and prior knowledge of the decision problem—impact predecisional information search and strategy use (Zakay, 1990; Shanteau, 1992; for recent work see Pachur and Marinello, 2013). Later, interest grew in the relation between strategy use and motivational characteristics such as need for cognitive closure (Shiloh et al., 2001; Vermeir et al., 2002), need for cognition (Verplanken, 1993), decisiveness and indecisiveness (Ferrari and Dovidio, 2001; Anderson, 2003). Also, cognitive ability was found to impact strategy selection (Bröder, 2003; Fasolo et al., 2003; Mata et al., 2007, 2010). Numerous task and context determinants of predecisional information processing and strategy use studied so far include framing of a decision task (Tversky and Kahneman, 1981), response mode (Tversky et al., 1988), type of learning task (Pachur and Olsson, 2012), information cost and information format (Bröder, 2000; Newell and Shanks, 2003); task complexity (Payne, 1976), time pressure (Wright, 1974; Rieskamp and Hoffrage, 1999), and many others (for overviews see Payne et al., 1993; Gigerenzer and Gaissmaier, 2011).

Notably, very few studies looked at how emotional factors influence predecisional information processing and strategy use. Mano (1992) reported that participants under social stress were more extreme in their judgments of job applicants, suggesting that they may have focused on only a subset of the presented information. Similarly, Lewinsohn and Mano (1993) showed that highly aroused participants acquired less information and did so in a more selective manner by focusing more on subjectively important attributes. Scheibehenne and von Helversen (2015) looked at how positive and negative mood states impact strategy selection, assuming that positive mood broadens the attention focus and negative mood narrows it. In line with this assumption they found that positive mood led to the more frequent use of compensatory strategies, whereas negative mood led to the more frequent use of simple noncompensatory heuristics. Wichary et al. (2015a) looked at how emotional arousal impacts the use of decision strategies. In that study, we used skin conductance as an index of autonomic arousal induced by the aversive and neutral pictures, in order to better control participants' state and to gain some insight into the neural correlates of predecisional information processing. We found that our manipulation led to reduced information search and increased allocation of attention to the most important cue. We also found that emotional arousal indexed by skin conductance leads to the use of simpler decision strategies. This pattern of results favors the attention narrowing hypothesis, which suggests that high arousal can lead to attention narrowing and in consequence, to the reliance on simple decision heuristics. The association of strategy use with skin conductance is also a hint at the neural underpinnings of strategy use, both peripheral and central. Our results indicate that the sympatho-adrenergic-medullary system (SAM; Schommer et al., 2008) is involved in decision processes under stress, as well as that the central structures associated with SAM activity might be involved, namely the anterior cingulate cortex (Critchley, 2005) and the neuromodulatory noradrenergic system (Aston-Jones and Cohen, 2005; Nieuwenhuis et al., 2011).

## Neural underpinnings of decision strategy selection

Insights into the neural mechanisms of strategy selection come from a separate line of research within neuroscience. Studies on multi-attribute choice have been reviewed by Krawczyk (2002); Venkatraman and Huettel (2012) and Volz and Gigerenzer (2012). These papers reviewed empirical evidence for the neural mechanisms of multi-attribute choice, however they did not focus on strategy use. Here, we focus on the use of compensatory and noncompensatory strategies, in situations when both kinds of strategies can be used, that is when knowledge beyond option recognition is available. Therefore, we did not include studies on the recognition heuristic by Volz et al. (2006) and Rosburg et al. (2011), because these studies analyzed situations when only recognition heuristic could be applied (or not) and did not look at the problem of selecting a strategy from a broader repertoire.

Several studies have attempted to identify the underlying neural sources of interindividual variability in strategy use. Venkatraman et al. (2009a), studying choices under risk using fMRI, showed that individual variability in the use of compensatory and simplifying decision strategies could be predicted by activity in ventral striatum, suggesting that high activity in the dopaminergic system might underlie the tendency to use the simplifying noncompensatory strategies. Moreover, Venkatraman et al. (2009b) provided evidence that strategic control of decisions is associated with the activity of anterior dorsomedial prefrontal cortex (DMPFC, see also Venkatraman and Huettel, 2012, for a review). Similarly, Gluth et al. (2013a), studying inference based multi-attribute choice with fMRI, showed the association between strategy selection and the activity of ventral striatum, the ventromedial prefrontal cortex and the anterior cingulate cortex. Khader et al. (2011) using a training paradigm and fMRI recording during a memory-based multi-attribute choice, showed that the amount of information that was required to use in a decision was reflected by the activity of left dorsolateral prefrontal cortex (DLPFC), and this activity modulated the activity in posterior areas associated with storage of decision cues. Replicating and extending these findings, Khader et al. (2015) showed that DLPFC activation is sensitive to both the number of decision cues that is retrieved in a controlled manner and the number of cues that are automatically activated by a decision option, suggesting that DLPFC activation reflects a general retrieval effort. Khader et al. (2011, 2015) also found that activation in the parietal cortex increased as a function the number of attributes to be retrieved. Together, these findings constitute important evidence for the involvement of the frontoparietal network in predecisional information processing.

## Cognitive models of strategy selection

The problem of using the right strategy for a particular decision task is framed as the *strategy selection problem* and several models have been proposed to account for this process (Table 2). The earliest are Beach and Mitchell (1978), Christensen-Szalanski (1978) and Payne et al.'s models (1988, 1993), which take a top-down approach to strategy selection. Regardless the differences, these models commonly assume that along with the repertoire of strategies, decision makers possess a priori knowledge of the cost and benefits of using a particular strategy, and integrating this knowledge leads to a (presumably) conscious, deliberate choice of a strategy. Strategy selection learning theory (SSL, Rieskamp and Otto, 2006), like the previous models, assumes that people possess a repertoire of strategies from which they can choose the appropriate strategy. It postulates that reinforcement learning of expectations of strategies' performance is the main driving force behind the tendency to use a particular strategy.

**Table 2:** **Cognitive models of decision strategy selection**.

**Model**	**Mechanism of selection**	**Approach**
Beach and Mitchell (1978), rule based model	Selection based on cost-benefit calculations	Top-down
Christensen-Szalanski (1978), rule based model	Selection based on cost-benefit calculations	Top-down
Payne et al. (1993), rule based model	Effort-accuracy calculations	Top-down
Strategy Selection Learning theory (SSL; Rieskamp and Otto, 2006), reinforcement learning model	Reinforcement learning, updating of strategy expectancies	Top-down
Lee and Cummins (2004), evidence accumulation model	Sequential sampling of evidence; variable evidence threshold	Bottom-up
Bergert and Nosofsky (2007), evidence accumulation model	Sequential sampling of evidence with attribute weights as free parameters and final choice as probabilistic	Bottom-up
Glöckner and Betsch (2008), connectionist model	Activation of network nodes, weighted summing of activations; parallel constraint satisfaction; network consistency compared to threshold	Bottom-up

Another model is Lee and Cummins' Evidence Accumulation Model (EAM, Lee and Cummins, 2004), which assumes that both the rational decision strategy (RAT) and the fast and frugal TTB strategy are special cases of a sequential sampling decision process. Thus, it is a rather radical departure from the previous models, because the assumption of the repertoire of strategies is absent in this model. In turn, its main assumption is that the TTB strategy and the rational strategy can be unified within one process. Building on EAM, Bergert and Nosofsky (2007) also proposed that TTB strategy and the rational model can be unified within one framework. The crucial difference was that Begert and Nosofsky relaxed several assumptions of the earlier model in order to make it more psychologically plausible, e.g., they assumed that decision makers do not always use the optimal attribute weights and that the final choice is not made deterministically, but rather in a probabilistic manner. Glöckner and Betsch (2008) proposed a connectionist, parallel constraint satisfaction model of predecisional information processing. Similarly to the above models, their approach is also based on a unifying mechanism. Their model proposes that probabilistic decision tasks can be represented as simple neural networks (Glöckner and Betsch, 2008). It assumes that the activation spreading through such networks and eventually settling in a balanced state is the underlying mechanism of choice. The settled state of network activation represents the state of maximum consistency, which is compared to a threshold, and when the given threshold is exceeded, the choice is made.

In sum, the strategy selection problem was first approached from the perspective of top-down models, assuming a repertoire of strategies that are themselves selected by a meta-mechanism that takes into account situational factors and decision maker's resources. Departures from this line of reasoning were bottom-up models which attempted to explain the apparent variability in strategy use with unifying mechanisms. Together, these models offered some insights into the cognitive processes underlying decisions. However, they did not offer any mechanistic explanation of how the model parameters might be changed. In other words, these models were not well grounded in theories of elementary cognitive processes. This opens the way for more explicit accounts of how strategy selection is shaped. We believe that further unified models should be proposed, grounded in empirical and theoretical work on attention, and in the neurophysiological work on the neural substrates of attentional processes. Since the evidence for the neural underpinnings of strategy selection is growing, these models should be designed as neurocognitive rather than purely cognitive models. Moreover, since the evidence for the impact of emotions on strategy use is also growing (e.g., Scheibehenne and von Helversen, 2015; Wichary et al., 2015a) and neural theories of how emotions impact cognition are available (Aston-Jones and Cohen, 2005; Mather et al., 2015), such models should incorporate emotional factors and their underlying neural substrates to fill the existing gap in explanation of how emotions impact decision making.

## Toward a neurocognitive model of decision strategy selection

In the following sections, we present a neurocognitive model of predecisional information processing and decision strategy selection. In order to go beyond empirical data and to understand the cognitive processes fully it is important to test mechanistic process models that try to explicitly state the underlying neurocognitive mechanisms (cf. Wichary et al., 2015b; Chuderski and Smolen, 2016). Within the domain of strategy selection in multi-attribute choice, there exists an empirical challenge described by Newell (2005) that it is impossible to distinguish, based on behavior, the accuracy of the unified and multiple strategy models described in the previous section. Therefore, we think it is time to build models of decision strategy selection that can provide predictions that go beyond behavior and that can be tested in neurophysiological studies. The need to develop such models is also underlined by the fact that several neurophysiological models of multi-attribute choice already exist (Louie et al., 2013; Chau et al., 2014; Hunt et al., 2014; Tsetsos et al., 2016). These models are relevant here, because they stress the importance of brain-wide computational processes as determinants of choice—particularly, the divisive normalization, hierarchical inhibition and gain control processes. Our model shares these computational principles and applies them to strategy selection.

The model, BUMSS (Bottom-Up Model of Strategy Selection), is based on our previous attempts to understand strategy selection in a unified, bottom-up manner (Smoleń and Wichary, 2008; Wichary and Smoleń, 2013). In these early version, as well as in the current version, the basic assumption is that in every choice situation people attempt to compute a weighted additive evaluation of decision alternatives. With this assumption, the question arises how to shape this general underlying process so that it resembles information processing with a simple noncompensatory heuristic at times. The idea behind our model is based on the observation by Martignon and Hoffrage (1999) that the TTB heuristic is equivalent to the WADD rule with a noncompensatory set of cue weights. A noncompensatory set of cue weights is a J-shaped distribution of cue weights, where one cue has a much higher weight than the other cues, such that the sum of their weights is not higher than the weight of the best cue. In contrast, a compensatory cue weight distribution is such that the cue weights are similar, and the sum of the less valid cues is higher than the weight of the best cue (Martignon and Hoffrage, 1999, 2002). In this perspective, the main question that our model tries to answer is how to obtain a noncompensatory set of cue weights using neurally plausible computations.

To illustrate the problem, consider a two-alternative, six-attribute choice task. The goal of the decision maker is to choose the more expensive diamond from the set of two. Each of the diamonds is described with six cues (attributes): size, overall proportions of the diamond, crown proportions, pavilion proportions, size of table and color (e.g., Mata et al., 2007). Each cue has a utility. In studies on multi-attribute choice, utility of a cue is often defined by the experimenter as cue validity—the conditional probability that a choice based on this cue is correct, given that the cue discriminates between the choice alternatives (Rieskamp and Hoffrage, 1999; Martignon and Hoffrage, 2002). There are many alternative measures that capture the utility of a cue, e.g., bayesian validity (Lee and Cummins, 2004), usefulness (Newell et al., 2003), success (Newell et al., 2004). Also, subjectively, cue utility can be determined by the decision maker through a host of reinforcement learning and memory processes that reflect how well the cue performed in the past and how relevant the cue is to the current goals set by the decision task (e.g., Rakow et al., 2005). The different definitions of cue utility and the processes that compute it are beyond the scope of the current paper. However, in many experiments on inference based multi-attribute choice, cue validity is explicitly given to the participants as a number, and participants use this information to guide their information acquisition and choice (e.g., Rieskamp and Hoffrage, 1999, 2008; Bröder, 2000, 2003; Newell et al., 2003; Newell and Shanks, 2003). In the current model, we stay with this conceptualization and assume that cue validities (*Q*) are available to the decision makers as the following numbers: 0.706 (size), 0.688 (overall proportions), 0.667 (crown proportions), 0.647 (pavilion proportions), 0.625 (size of table), and 0.6 (color). Each cue takes one of the two values for each diamond: low (0) or high (1) (see example in Table 3).

**Table 3:** **Example of a multi-attribute choice task with validities, values, and weights of the cues for low (5) and high (35) values of the inverse temperature parameter β**.

	**Cue**					
	**1**	**2**	**3**	**4**	**5**	**6**
Cue validity	0.706	0.688	0.667	0.647	0.625	0.6
Cue values for alt. 1	1	0	0	0	0	1
Cue values for alt. 2	0	1	0	1	0	1
Cue weight (β = 5)	0.211	0.193	0.174	0.157	0.141	0.124
Cue weight (β = 35)	0.501	0.267	0.128	0.063	0.029	0.012

**Figure 1:**
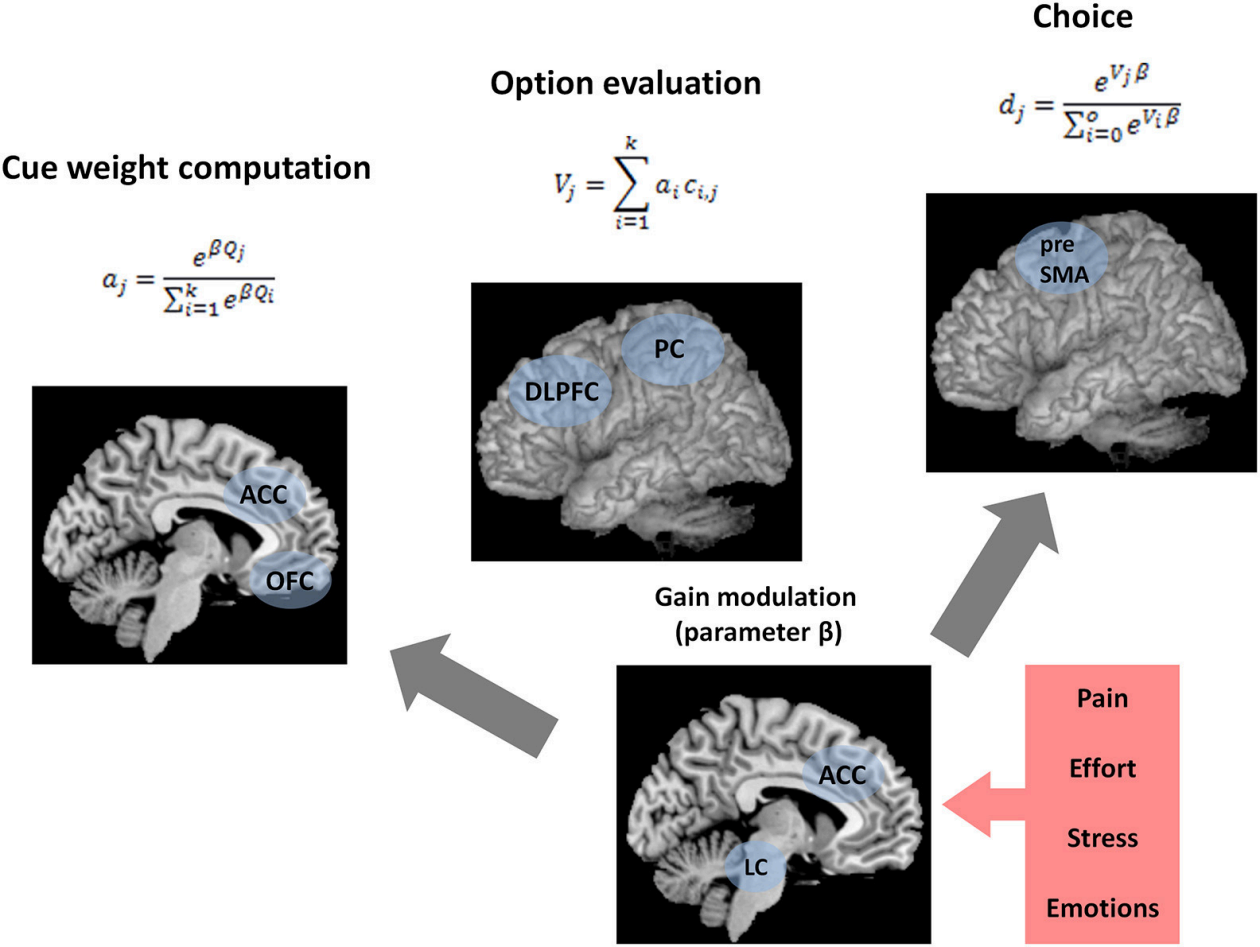
**Summary of the bottom-up model of strategy selection (BUMSS)**. Computations and brain structures postulated by BUMSS as the mechanism of decision strategy selection and choice. For any multi-alternative, multi-attribute choice task, first, the attention weights (*a*) of the attributes are computed, by the orbitofrontal cortex (OFC) and anterior cingulate cortex (ACC), on the basis of the initial cue validities (*Q*) (**left panel**). During this process, the phasic gain modulation (change in β) mediated by locus coeruleus (LC) increases the attention weights of the most valid attribute while decreasing the weights of the other attributes. These attention weights enter the option evaluation process (**middle panel**). For each option, its evaluation is computed as the summation of all attribute values multiplied by their attention weights. This is computed by the frontoparietal network, consisting of the dorsolateral prefrontal cortex (DLPFC) and the parietal cortex (PC). Finally, option evaluations determine the probabilities of choosing the options (**right panel**), a process performed by presupplementary motor area (preSMA). These probabilities are also influenced by phasic gain modulation by LC. Gain modulation is affected by ACC activity, which is modulated, in turn, by affective context: pain, effort, stress, and emotions.

**Figure 2:**
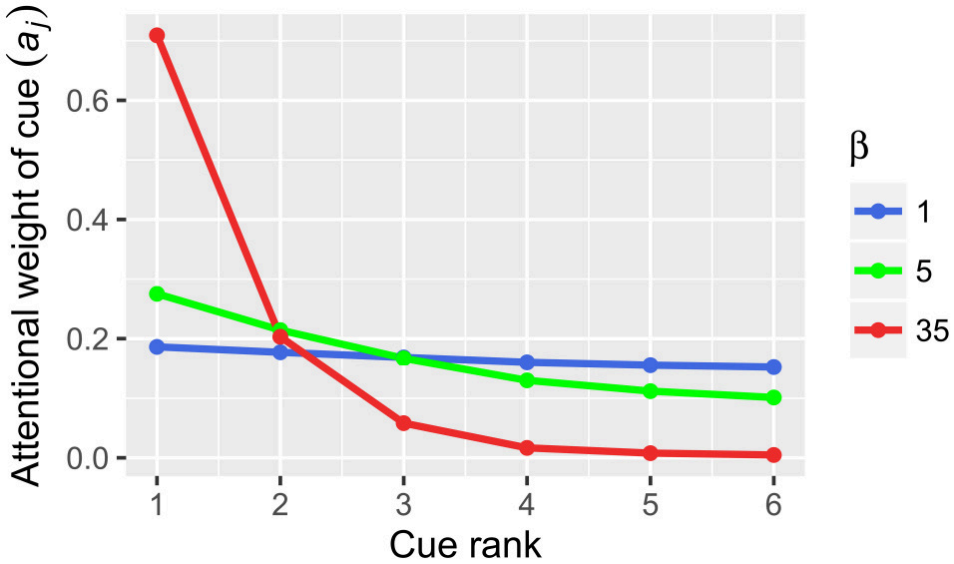
**Cue weights computed from cue validities by Equation (1) in BUMSS**. In our example, the initial cue validities (*Q*): 0.706, 0.688, 0.667, 0.647, 0.625, 0.6 are transformed by the softmax rule into the cue weights (*a*). With low values of the inverse temperature parameter β (1, 5), the resulting cue weights have a compensatory distribution, similarly as the original cue validities. With a high value of the β parameter (35), the initial compensatory distribution of cue validities results in a noncompensatory distribution of cue weights.

According to BUMSS, there are three processes that form the bottom-up mechanism of decision strategy selection and lead to the final choice: (1) cue weight computation, (2) gain modulation, and (3) weighted additive evaluation of alternatives (Figure 1). BUMSS postulates that in order to choose one alternative from several alternatives, the decision maker will first acquire and then weigh available decision cues, according to the softmax rule (Sutton and Barto, 1998; Doya, 2002), which is an example of divisive normalization (Louie et al., 2013):

(1) $$\begin{array}{r} a_{j}=\frac{e^{\beta Q_{j}}}{\sum_{i\;=\;1}^{k}e^{\beta Q_{i}}} \end{array}$$

where *a*_*j*_ is the weight of decision cues *j, Q*_*j*_ is the *cue validity* of cue *j* and β is the gain control parameter. The crucial parameter in Equation (1), β (*inverse temperature)*, reflects the changes in neural gain (e.g., Doya, 2002; Aston-Jones and Cohen, 2005). In computational neuroscience terms, gain is the amplification of neural activation, which is achieved by additional input into a signal processing pathway and is usually expressed as multiplication of the signal (Eldar et al., 2013; Louie et al., 2013). According to Equation 1, changes in gain lead to the changes in cue weighting (Figure 2). In our example, gain increase from 1 to 35 will transform the original cue validities (0.706, 0.688, 0.667, 0.647, 0.625, and 0.6) with a compensatory distribution into the resulting cue weights (0.501, 0.267, 0.128, 0.063, 0.029, 0.012) with a noncompensatory distribution (Table 3). Note that in our example such a noncompensatory distribution is only obtained with a very high value of β (35), whereas small values of β (1 or 5) lead to compensatory distributions of cue weights (Figure 2).

After acquiring and weighting the decision cues, the decision maker will then attempt to evaluate decision alternatives by integrating the weights and values of the decision cues, which is described by the weighted additive rule:

(2) $$\begin{array}{r} V_{j}\;=\sum_{i\;=\;1}^{k}a_{i}\;c_{i,j} \end{array}$$

where *V*_*j*_ is the value of a choice alternative *j, a*_*i*_ is the cue weight and *c*_*i, j*_ is the value of *i*th cue for *j*th alternative. In our example, the option values are *V*_1_ = 0.335, *V*_2_ = 0.474 for β = 5 and *V*_1_ = 0.513, *V*_2_ = 0.342 for β = 35, thus showing that different values of β parameter can lead to qualitatively different option evaluations. Finally, the decision maker will choose an alternative *j* from several alternatives in a probabilistic manner, with a probability *d*_*j*_ that is given again by the softmax rule (Sutton and Barto, 1998; Doya, 2002):

(3) $$\begin{array}{r} d_{j}\;=\;\frac{e^{V_{j}\beta }}{\sum_{i\;=\;0}^{o}e^{V_{i}\beta }} \end{array}$$

where *V*_*j*_ is the value of alternative *j, o* is the number of options and β is the same inverse temperature parameter as in (1) that reflects the gain and in consequence, controls the stochasticity of the choice. For increasing β, the probability that the alternative with the highest value will be chosen, increases. For a choice between two alternatives *V*_1_ and *V*_2_, this will be shown as changes in the steepness of the well-known sigmoid function (Figure 2). In our example, with the low values of β (in both Equations 1 and 3), the probabilities for alternative 1 and 2 will be equal to 0.333 and 0.667, respectively. With high values of β, the probabilities will be equal to 0.997 and 0.003, respectively. Note the influence of β on this stage. Although the absolute difference between the values of the alternatives computed by Equation (2) was similar in both cases (0.14 and 0.17) the probabilities of choice differ substantially for different values of β.

## How is BUMSS implemented in the brain?

Apart from specifying the computational processes, the aim of the current model is to propose a set of neural substrates underlying choices consistent with WADD and TTB. We postulate that the computational processes described above take place in a brain network consisting of (1) the dorsolateral prefrontal cortex (DLPFC) and parietal cortex (PC)—which form the fronto-parietal network (FPN) and are responsible for cue weight computation and weighted additive evaluation, (2) the orbitofrontal cortex (OFC), which contributes to cue weight computation, (3) the anterior cingulate cortex (ACC), and (4) the brainstem nucleus locus coeruleus (LC), which contribute to cue weight computation and choice by modulating gain (Figure 1).

Fronto-parietal network, particularly its components the dorsolateral prefrontal cortex (DLPFC) and the parietal cortex (PC), performs the operations of maintaining and integrating information necessary for current and future behavior and thus is the neural substrate for working memory processes (Linden et al., 2003; Erikson et al., 2015). Orbitofrontal cortex (OFC) computes positive and negative utility of incoming stimuli (Tremblay and Schultz, 2000; Aston-Jones and Cohen, 2005; Padoa-Schioppa and Assad, 2006; Wallis, 2007; Volz and von Cramon, 2009). Anterior cingulate cortex (ACC) serves a broad range of functions (Paus, 2001) such as conflict monitoring, cognitive control (Botvinick, 2007), and regulation of autonomic activity (Critchley, 2005). Growing evidence suggests that it computes negative utility or cost of the undertaken actions (Aston-Jones and Cohen, 2005). Although evidence suggests that OFC and ACC may both code positive and negative utility, within the current model, we stay with the Aston-Jones and Cohen's (2005) simplifying assumption that the OFC codes the positive utility of incoming stimuli and the ACC encodes the negative utility of engaging in a given behavior.

ACC receives multiple inputs from various brain structures: prefrontal cortex, parietal cortex, amygdala, insula and somatosensory cortex, among others (Paus, 2001). Inputs from the PFC relay the information on the information processing demands associated with performing a current task (Paus, 2001). Inputs from the parietal cortex, mainly the temporo-parietal junction (TPJ), relay the information about stimuli salience, therefore the ACC (together with the TPJ, insula and locus coeruleus) is involved in computing cue salience (Menon and Uddin, 2010; Litt et al., 2011; Kahnt and Tobler, 2013; Kahnt et al., 2014). In addition, inputs from the amygdala, insula and somatosensory cortices relay information about threatening or noxious stimuli to ACC (Bornhövd et al., 2002; Kuo et al., 2009; Etkin et al., 2011). It is important to note that ACC activity is modulated by a wide range of experimentally manipulated variables, including pain, threatening stimuli, conflicting cues, physical and cognitive effort (Shackman et al., 2011) and thus can serve as an integrated representation of the noncognitive context of decision processes.

Brainstem nucleus locus coeruleus is involved in regulating arousal and attention, the sleep-wake cycle and the stress response. It is a main source of brain norepinephrine (NE). LC neurons respond in a tonic and phasic manner to aversive as well as appetitive stimuli and release NE that mediates the mobilization of energy for avoiding aversive stimulation or obtaining a reward (Rajkowski et al., 2004; Aston-Jones and Cohen, 2005; Sara and Bouret, 2012). Importantly, NE release regulates the gain of information processing in the cortex. LC performs this by widespread connections with most of the brain. It has reciprocal connections with the dopaminergic system that implicate LC in mobilization for reward processing (Varazzani et al., 2015). Both OFC and ACC have strong reciprocal connections with LC (Aston-Jones and Cohen, 2005) and OFC may relay its information about stimulus utility via ventral striatum (Ullsperger et al., 2014). LC is connected to FPN, where it projects its diffuse connections which are the basis for cortex-wide gain modulation (Eldar et al., 2013). LC is also reciprocally connected to amygdala, which regulates LC function by corticotrophin releasing hormone (CRH, Owens and Nemeroff, 1991). Similarly, LC is reciprocally connected to hypothalamus (and thus to the whole hypothalamic-pituitary-adrenal axis), which activates it by CRH (Owens and Nemeroff, 1991). Through a common afferent, the nucleus paragigantocellurlaris, LC is also connected to the sympathetic-adrenal-medullary (SAM) system and thus it is co-activated with the peripheral effectors of the autonomic nervous system responsible for the galvanic skin response or pupil dilation (Nieuwenhuis et al., 2011).

### Cue weight computation and weighted additive evaluation of alternatives

BUMSS assumes that prior to making a multi-attribute choice, the decision maker acquires the available information in a sequential manner. First, when a cue enters the cortex, its validity and salience are determined by OFC and the salience network (including the ACC), based on past associations and physical features. If a cue is important due to its goal relevance, past performance or physical characteristics, it elicits high activation of OFC and ACC. OFC and ACC relay their activations to locus coeruleus, and the phasic OFC and ACC activation elicits phasic LC response. This LC phasic response is the neural substrate of the momentary gain modulation that contributes to the final cue weight computation—a process where the incoming decision cue is weighted, i.e., deemed important and worth further processing or deemed unimportant and worth ignoring.

The model assumes that FPN holds the incoming cues and integrates them into the weighted additive evaluations of alternatives. The anterior part of the FPN, the DLPFC, is activated by the incoming decision cues, but it is also activated by other stimuli that might act as distracters (Khader et al., 2015). Thus, in the current model, DLPFC activation reflects the general effort of processing information. We assume that this activation, driven both by relevant stimuli and distracters, is passed down to ACC, which in turn activates LC, increasing its tonic activity. In other words, besides taking part in the phasic responses to cues, ACC also codes the effort of processing the information in working memory. We assume that with each cue incoming to the FPN, ACC is more active. However, ACC activation may depend on the efficiency of DLPFC in handling the cues. If an individual's DLPFC is efficient in maintaining stored items, then the same number of processed cues activates ACC to a lesser extent than in the case when DLPFC is not efficient. When DLPFC is processing many cues simultaneously, it highly activates ACC. ACC receives this information and also receives the information regarding physical effort, pain, and other states of negative utility (hunger, thirst, negative emotions, Aston-Jones and Cohen, 2005), serving as a hub for coding negative utility from different domains and modalities.

### Gain modulation

As already mentioned, BUMSS assumes that ACC activates locus coeruleus—with increasing ACC activation, LC tonic activity increases. According to the *adaptive gain control theory* (Aston-Jones and Cohen, 2005), the relationship between LC-NE tonic activity and phasic gain changes is curvilinear (inverted-U). When the LC-NE tonic activity is low, LC-NE phasic response to cues is weak (gain increase is small). When the tonic LC activity is moderate, its phasic response is strong (gain increase is high), and again when the tonic activity is high, the phasic response is low (gain increase is small). Increases of tonic activity from low to moderate cause the increase in gain for incoming information, whereas increases from moderate to high tonic activity cause decreases in gain to incoming information.

One of the characteristic features of the FNP, important for the current model, is lateral inhibition. It is one of the mechanisms underlying attentional selectivity, as postulated in several models of attention (Walley and Weiden, 1973; Itti et al., 1998; Edin et al., 2009; Markovic et al., 2014; Mather et al., 2015) and has been already implemented in models of value-based choice (Louie et al., 2013; Hunt et al., 2014). Particularly, the model by Mather et al. is important here, because it offers an explicit mechanistic account of the attentional effects of norepinephrine in the cortex, that are based on inhibition. The model GANE (Glutamate Amplifies Noradrenergic Effects) posits that norepinephrine acts together with glutamate to form activation hot spots in the cortex that represent the stimuli prioritized by attention. In accordance with GANE, our model postulates that phasic gain increases mediated by LC and the lateral inhibition within FPN modulate attentional selectivity such that highly activated salient cues inhibit the activation of less salient cues. Within our model, this leads to the processing of only single, best cues and thus results in the application of the Take The Best strategy to the multi-attribute choice problem.

As said above, we assume that the increased ACC activity drives LC tonic activity. And in turn, increased LC tonic activity leads to the change in phasic LC responding. The exact direction of this change (increase or decrease) depends on the initial, prestimulus level of LC tonic activity (Aston-Jones and Cohen, 2005). If the tonic LC activity is initially low, then ACC drive will increase it to the moderate level, which will lead to increased phasic responses to incoming decision cues. This will lead to better gating of these cues into the FPN and, on the psychological level, their higher impact on the option evaluation. If the tonic LC activity is moderate, then its further increases by ACC drive will lead to weaker phasic responses to incoming cues, and, as a consequence, their poorer gating into FPN and lower impact on the option evaluation.

**Figure 3:**
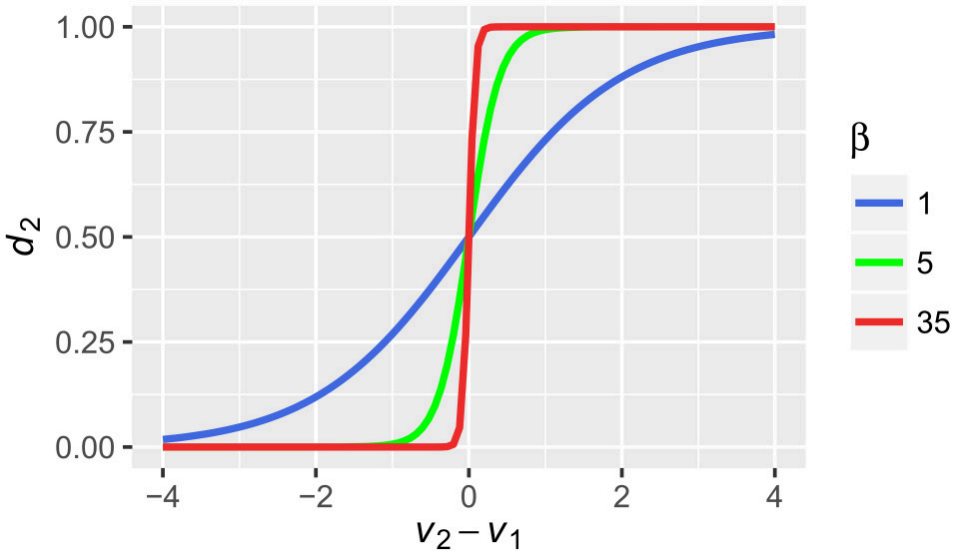
**The relation between probability of chosing alternative 2 (*d*_2_) based on the integrated values of alternative 1 and 2 (*V*_1_, *V*_2_) that were transformed by the softmax rule with different values of the inverse temperature parameter β**.

The current model postulates that in order to make a multi-attribute choice, the decision maker will try to make the final decision by integrating the cues. Cues that have high gain (are salient), will be better represented in working memory and will weigh more on the final evaluation of the choice options. In contrast, cues that have low gain (are not salient), will be poorly represented in working memory, and will weigh less on the final evaluation of the choice options. In extreme cases, when the tonic LC activity is very high and only the first cue is able to produce any phasic response, and the following cues do not produce any phasic response from LC, only one cue is represented in working memory and is the basis for the final choice. Within the current model, this is akin to using the lexicographic Take The Best heuristic. Finally, the model postulates that the FPN computes the values of the choice alternative and relays these computations to the premotor areas and the cortico-striatal networks that perform action selection (Bogacz and Gurney, 2007; Forstmann et al., 2008; Gluth et al., 2013b). The model assumes that this final stage can also be influenced by gain modulation (Equation 3), so that under high LC activity, the difference between the valuations of the alternatives is enhanced, further biasing the choice process toward the alternative with the higher value (Figure 3). Given the mechanism specified above, we can understand how various situational factors might impact decision strategy use and lead to the use of simple, boundedly rational heuristics. In short, the answer is that various factors that drive ACC activity, such as cognitive load, stress or pain, will increase tonic LC activity and this will change the gain of cortical information processing in the frontoparietal network that leads to selective processing of decision cues and to one-reason decision making.

## Discussion and conclusions

The aim of this paper is two-fold: first, to review the existing evidence on decision strategy use, the cognitive models and the neural underpinnings of decision strategy selection and second, to propose an original neurocognitive model of this process, with a particular focus on the role of noncognitive factors in shaping strategy selection. To this end, we reviewed the relevant literature on strategy selection and identified gaps in our knowledge of factors influencing this process. Particularly, only a limited number of studies took into account the impact of emotional factors on predecisional information processing and decision strategy use. These gaps suggest that further studies are needed in this domain. The review of the existing models of decision strategy selection also pointed out that most of the existing models are top-down, that is they assume a master, metacognitive process that selects among the decision strategies available to the decision maker. This feature of the top-down models leads to one well known problem: the need for a metastrategy to select among the individual strategies which eventually leads to the regress to infinity (e.g., Newell, 2005). Another serious problem of the top-down frameworks is strategy sprawl, that is constant adding of new heuristics to the repertoire (Scheibehenne et al., 2015).

Such limitations led to the formulation of unifying models which attempted to reduce the multiplicity of heuristics to more general information processing mechanisms. Our model presented in this paper is another proposal how to unify the use of the rational, compensatory decision strategies with the noncompensatory, one-reason decision heuristics. It is closest to Lee and Cummins (2004) and Bergert and Nosofsky (2007) models, however it goes beyond these models. This model attempts to reduce the apparent variability of decision strategies by offering a unifying mechanistic account that is rooted in neurophysiology. Thus, it offers a mechanistic neural processing account of decision strategy selection. Moreover, it allows for incorporating noncognitive factors that may influence strategy selection, such as emotional arousal, stress and effort. It does so by explicitly linking higher level cognitive processes that take place in cortical areas with brainstem activity, and shows how this activity can shape decision strategies and eventually, choices. Because the territory of how emotions impact decision making is rather uncharted, our model also offers some of the first mechanistic insights how these processes might be operating.

### Relations to other models

Our model is focused on explaining decision strategy selection. However, it draws from other models, formulated in various domains. In terms of computational assumptions, our model is similar to neuroscientific models explaining option selection in value-based choice (Louie et al., 2011, 2013; Chau et al., 2014; Hunt et al., 2014; Tsetsos et al., 2016). Our model shares computational assumptions with models by Hunt et al. (2014) and Louie et al. (2013). Particularly, by employing the softmax rule for the cue weight computation and option selection, our model relates to the divisive normalization process, postulated by Louie et al. (2013) as a general brain mechanism for value computation. The current paper shows that divisive normalization and gain modulation can be applied to understand not only option selection, but also decision strategy selection. In this view, the simplification of decision strategy, from WADD to TTB, is a consequence of normalization process coupled with gain increase. This offers a possibility to combine the above models of option selection with the current model of strategy selection, in order to explain a greater range of choice phenomena.

As for the neural implementation of these computational processes, the findings by Chau et al. (2014); Hunt et al. (2014) and Louie et al. (2011, 2013), suggest an important role of intraparietal sulcus (IPS) in attribute relevance computation and dorsomedial prefrontal cortex (DMPFC) in integrated option value computation. Our model is broadly consistent with these findings. It postulates the role of ACC (part of DMPFC) and PC in strategy selection through the computation of attribute weight and integration of attribute values into an overall option value. In light of such results, our model could be refined to postulate IPS as a node for attribute weight computation.

Our model offers a possibility to understand how the process of strategy selection is shaped by the affective context, which can be conceptualized as the impact of arousal on strategy selection and which has been observed in our previous studies (Wichary and Rieskamp, 2011; Wichary et al., 2015a). To explain this phenomenon, our model explicitly postulates an important role of the brainstem neuromodulatory locus coeruleus in strategy selection. In this, it draws from the adaptive gain control theory (AGT) and from the anatomical and neurophysiological work on the norepinephrine system (Aston-Jones and Cohen, 2005). It also draws from the recent GANE model of the effects of norepinehrine and glutamate on information processing in the cortex (Mather et al., 2015), which might be viewed as a detailed extension of the AGT.

BUMSS connects these models with traditional models of choice, present in the decision making and economic literature, namely the weighted additive evaluation (e.g., Keeney and Raiffa, 1993). This link is possible through conceptualizations that are also present in Lee and Cummins (2004) and Bergert and Nosofsky (2007) models of strategy selection, namely that there is one mechanism unifying the use of the rational and heuristic strategies and that this process can be related to how decision makers weigh incoming decision cues. More broadly, such conceptualizations are related to the general class of evidence accumulation models. The usefulness of these models for understanding decision strategy use has already been noted by Newell (2005) and Bröder and Newell (2008). Particularly, our model bears analogies to a version of evidence accumulation model with a narrowing threshold, proposed by Gluth et al. (2013b), where the evidence processed before a choice accumulates to a bound that decreases over time. Based on the evidence reviewed in this paper, we can propose a hypothesis that changes in arousal mediated by locus coeruleus, and the gain modulation associated with it, are the neural basis of such a narrowing decision threshold.

### How can the model be tested?

Our model allows to go beyond the purely cognitive and behavioral predictions regarding decision strategy use. It allows us to link the process of strategy selection in multi-attribute choice with the functioning of the arousal modulatory system and its peripheral physiological indices, such as skin conductance and pupil dilation, and with neuroimaging data, EEG and fMRI (Nieuwenhuis et al., 2005, 2011; Murphy et al., 2014). Our data (Wichary et al., 2015a) provide some indication that the relations postulated by the current model linking skin conductance and strategy selection are valid. Precise, computational work is needed to reconcile these data with the predictions of the current model. We predict that skin conductance should be correlated with the values of β parameter in the current model fitted to participants choice data—high skin conductance should be associated with high values of β and thus a noncpomensatory cue weight distribution and choices consistent with TTB heuristic. Another peripheral signal, pupil dilation response is also a valid index of LC function (Gilzenrat et al., 2010; Jepma and Nieuwenhuis, 2011; Murphy et al., 2014). Our current empirical work is focused on gathering data that link decision strategy use in multi-attribute choice with this index. We predict that large pupil dilations to the most valid decision cues will be associated with high values of β parameter and a greater reliance on the simple heuristic TTB. Also, EEG indices such as the P300 ERP and EEG oscillatory activity are other possible indices of LC functioning (Danysz et al., 1989; Berridge and Foote, 1991; Nieuwenhuis et al., 2005). Our current empirical work (Wichary et al., under review) suggests that P300 ERP component is a viable correlate of rational and heuristic strategy use. Our current computational work is focused on linking the predictions of the current model with these empirical data. Last but not least, direct measurement of LC activity with fMRI, although technically challenging due to small size of LC, is possible (Sasaki et al., 2006), and can be linked to choices consistent with WADD and TTB strategies. We expect that high LC activation evident in BOLD signal will be associated with high values of β parameter and high proportion of choices consistent with TTB heuristic.

### Limitations of the current model

As any theoretical model, the model presented here is a simplification of the truly existing relationships. Our model is rather narrow, in that it discusses only the relation between an extremely complex and an extremely simple strategy, the WADD normative rule and the TTB heuristic. We chose to focus on WADD and TTB, because empirical evidence suggests that together, these two strategies explain a large majority of choices in studies on multi-attribute decision-making (Rieskamp and Hoffrage, 1999, 2008; Bröder, 2000; Newell et al., 2003; Newell and Shanks, 2003). Importantly, we also focused on these two strategies in order to be able to offer a detailed mechanistic account of the process leading to strategy selection. Moreover, our model stresses the bottom-up perspective on decision strategy selection. This perspective assumes that attentional processes are important for shaping decision strategy use. However, the top-down perspective on strategy use must not be ignored. As pointed by Kruglanski and Gigerenzer (2011), strategy use may be shaped both by the bottom-up and top-down processes. Future neurocognitive models of multi-attribute choice will have to incorporate these two perspectives.

On the neuroscience side, the model is limited in that it omits several important anatomical structures. This is most notably the dopaminergic (DA) system, which is implicated in coding of positive utility, reward, and arousal, as well as in working memory processes (Schultz, 2007; Arnsten et al., 2012). DA system was also postulated to work together with NE system in gain modulation (Servan-Schreiber et al., 1990), however only recently this interplay has been fully addressed in empirical studies (Ullsperger et al., 2014; Varazzani et al., 2015). Future versions of the model should put more emphasis on the role of dopamine in shaping decision strategy selection under variable motivational circumstances.

Last but not least, the model presented here is a mathematical and verbal sketch rather than a fully developed computational model. As such, it requires further work, mainly translating the process descriptions and equations into neurally plausible computations. The ultimate goal of such a modeling exercise is to integrate models of decision strategy use in multi-attribute choice with models that make use of low level neural mechanisms (e.g., Usher and Davelaar, 2002; Zhang and Bogacz, 2010; Hunt et al., 2014). Since most traditional models of decision making ignore the impact of noncognitive (e.g., emotional) factors on decision making, our model is also an initial proposal how to incorporate such factors into models of multi-attribute choice, and thus it can contribute to our understanding of how emotions affect our decisions. We hope that the presented model will stimulate new empirical investigations and theories in the neuroscience of decision making.

## Author contributions

SW and TS developed the theoretical model, and wrote and edited the manuscript.

## Funding

This research was supported by the postdoctoral fellowship from the Copernicus Centre for Interdisciplinary Studies in Krakow, an intramural Grant No. WP/2015/A/25 from the University of Social Sciences and Humanities in Warsaw and a grant from the Ministry of Science and Higher Education of Poland (NN106219338) to SW.

### Conflict of interest statement

The authors declare that the research was conducted in the absence of any commercial or financial relationships that could be construed as a potential conflict of interest.
